# Palatal approach for surgical removal of mesioangularly impacted maxillary third molar - a pilot study

**DOI:** 10.1186/s12903-023-03234-1

**Published:** 2023-07-25

**Authors:** Rui Sun, Yu-qi Sun, Yu Cai, Jihong Zhao

**Affiliations:** 1 State Key Laboratory of Oral & Maxillofacial Reconstruction and Regeneration, Key Laboratory of Oral Biomedicine Ministry of Education, Hubei Key Laboratory of Stomatology, School & Hospital of Stomatology, Wuhan University, No. 237 Luoyu Road, Wuhan, 430079 China; 2 Department of Oral and Maxillofacial Surgery, School & Hospital of Stomatology, Wuhan University, No. 237 Luoyu Road, Wuhan, 430079 China

**Keywords:** Palatal approach, Impacted maxillary third molar, Impacted tooth extraction, Traumatic ulcers of the lips

## Abstract

**Objective:**

This study aimed to investigate the application of the palatal approach for surgical removal of IMTM, and to evaluate its success rate, surgical duration, postoperative outcomes, and incidence of complications.

**Method:**

Patients with mesioangularly IMTM (Archer Classification Class B) in the none-buccal position to the adjacent second molar, which were indicated for surgical removal, were enrolled in this study. The patients were assigned into two groups according to the surgical approach: the buccal or palatal approach. The impacted tooth positions, diagnosis, past dental and medical history, and radiographic examination were recorded pre-operatively. The duration, surgery details, and surgical complications were documented during the surgery.

**Result:**

40 teeth were enrolled in our study. All teeth were removed completely. The operation time was significantly shorter in the palatal approach group compared to the buccal approach group (13.3 ± 2.8 min vs. 22.3 ± 5.5 min, *P*<0.001). The incidence of traumatic ulcers of the lips was significantly higher in the buccal approach group than in the palatal approach group (7/20 vs. 0/20, *P* = 0.008).

**Conclusion:**

It is more efficient to perform surgery with a palatal approach if a Class B mesioangularly IMTM is located in the non-buccal aspect of the adjacent second molar.

**Clinical trial registration number:**

ChiCTR2000040063

## Introduction

Impacted maxillary third molar (IMTM) surgery is a complicated dentoalveolar surgery [1], which may lead to some serious complications [2–5]. As compared to those that are buccally erupted to the adjacent second molar, surgical removal of IMTM which are distally or palatally impacted to the maxillary second molar can be technically challenging. More commonly, the IMTM surgery is performed via a buccal approach. It is not difficult to expose and elevate the third molar which is buccally impacted to the adjacent second molar. However, the difficulty of the surgery increased if the IMTM were not located in the buccal position. The range of mouth opening may affect the positioning and use of surgical instruments, in which traumatic ulcers are more likely to occur in patients with limited mouth opening.

Indeed, even the latest review [6] and original articles [7, 8], which summarized the surgical removal of the impacted maxillary third molar, lacked discussion on treatment strategies for such cases. Thus, a more effective surgical approach should be improvised to reduce post-operative complications.

As CBCT provides better spatial information on the impacted teeth, it is necessary to evaluate the position between IMTM and the adjacent second molar pre-operatively. The relationship between the impacted maxillary third molar and the adjacent second molar was mostly described by orthodontists [9, 10], however, there are limited oral surgery publications available to discuss such issues. To our knowledge, there is no classification available yet to describe the buccal-palatal position of IMTM, particularly in cases that require surgical removal, and this classification should guide the clinicians in deciding the appropriate surgical approach.

In this study, we proposed the use of palatal approach in surgical removal of the Class B mesioangularly IMTM which is either distally or palatally impacted to the adjacent second molar. We aimed to investigate the effectiveness and safety of this strategy, by comparing it to the conventional buccal approach.

## Materials and methods

STROBE checklist was followed throughout in reporting this study. This study followed the Declaration of Helsinki on medical protocol and ethics and the study was approved by the Regional Ethical Review Board of the Ethics Committee of the Hospital of Stomatology, Wuhan University (NO2020-B72). The inclusion criteria included: (1) Archer Classification [11] Class B mesioangular IMTM: The occlusal surface of the impacted tooth is at the middle of the crown of the adjacent second molar; the mesial cusp of IMTM is located near the cervical line of adjacent tooth (2) teeth with complete root(s) formation; (3) The tooth was indicated for extraction; (4) the IMTM was in contact with, distally or palatally impacted to the adjacent second molar.

The exclusion criteria included (1) more than one-fourth of the crown erupted; (2) mobile or missing adjacent tooth; (3) the patient was pregnant; (4) patients with pre-existing trismus; (5) severe gag reflex; (6) past or present medical history of recurrent angular cheilitis; (7) refusal to cone-beam computed tomography (CBCT) examination; (8) other factors that cause poor compliance or cooperation.

### Preoperative assessment

**Fig. 1:**
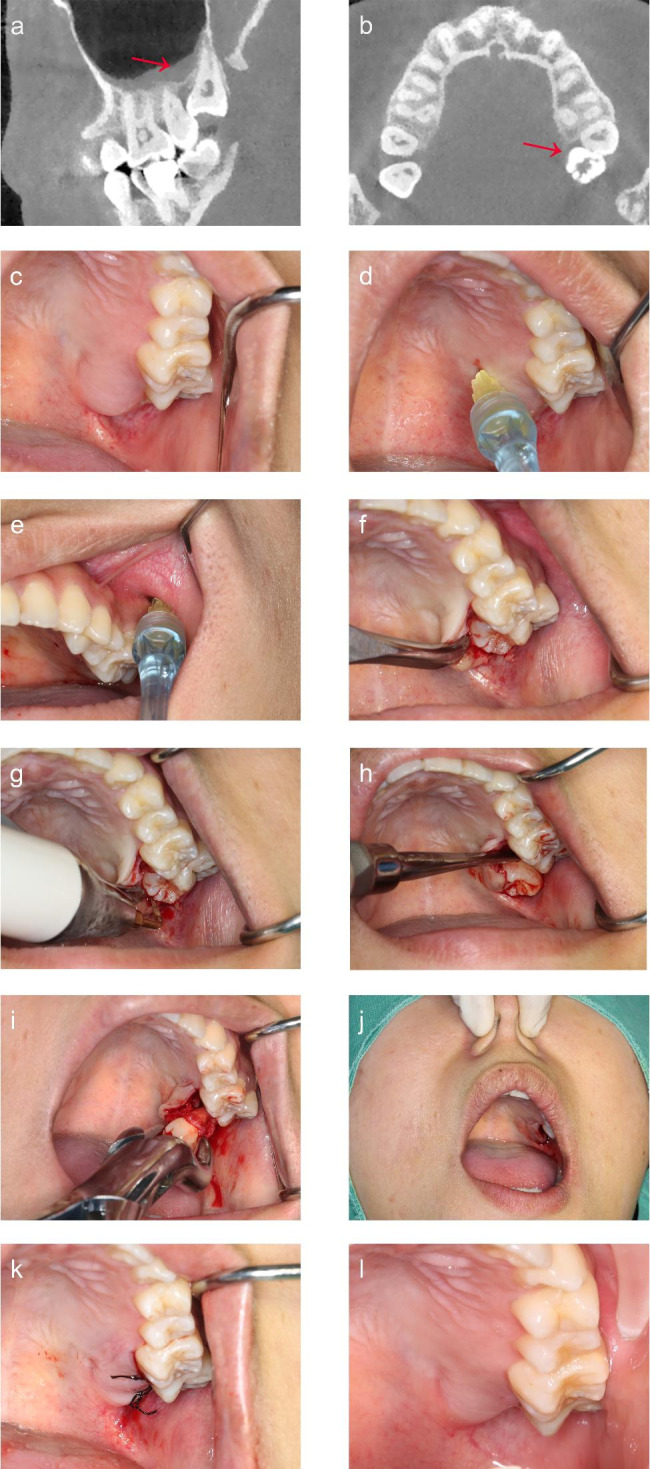
Extraction of maxillary impacted third molar in Class B located in the none-buccal position of the second molar. CBCT showed that the IMTM was mesioangular (**a**), and located in the palatal position of the second molar (**b**). The IMTM was not visible clinically(**c**). Greater palatine nerve block (**d**), and posterior superior alveolar nerve block were administered (**e**). The incision of the flap begins at the maxillary tuberosity and extends as far as the distal aspect of the second molar, continuing palatally along the cervical lines of the last two teeth, and ending at the medial aspect of the first molar. The mucoperiosteal flap was elevated to expose the impacted tooth(**f**). Piezosurgery was used for bone guttering to reduce the distal and palatal bone resistance(**g**). The tooth was luxated by dental elevator(**h**), and removed by a dental forceps (**i**). the socket was assessed for oroantral communication (if any) (**j**) and primary closure was done (**k**). The socket was healing in progress at one week post operation (l)

**Fig. 2:**
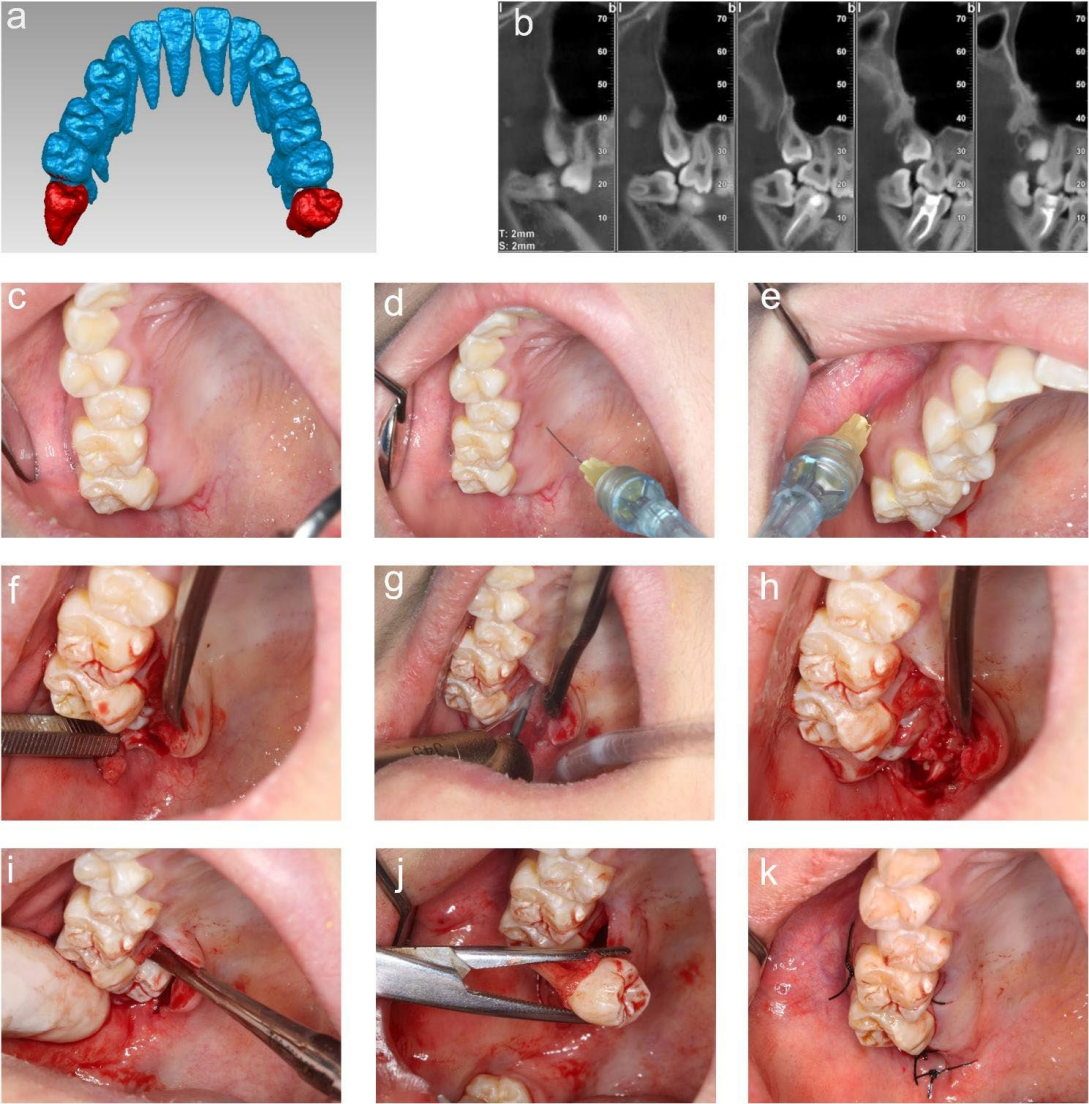
Extraction of maxillary impacted third molar in Class B located in the none-buccal position of the second molar. 3D reconstruction of maxillary teeth by software of DeepCare Dental AI, showing that the long axis of tooth 18 was palatally inclined (**a**). CBCT showed that the IMTM was mesioangular (**b**). The IMTM was impacted (**c**), Greater palatine nerve block (**d**), and posterior superior alveolar nerve block were given (**e**). The incision of the flap begins at the maxillary tuberosity and extends as far as the distal aspect of the second molar, continuing palatally along the cervical lines of the last two teeth, and ending at the medial aspect of the first molar. The mucoperiosteal flap was elevated to expose the impacted tooth(**f**). The fissure bur was used for bone guttering to reduce the distal and palatal bone resistance (**g**). After the bone resistance was removed (**h**), the tooth was luxated by dental elevator(**i**), and removed by dental forceps(**i**). The socket was assessed for any oroantral communication prior to wound closure (**k**)

CBCT is recommended to confirm the location of the tooth, the thickness of the alveolar bone around the IMTM, and the critical adjacent anatomical structure (the root of the second molar, the location of the floor of the maxillary sinus). (Fig. 1A &B). The image reconstruction was performed using the software, DeepCare Dental AI (Beijing, China) for clearer visualization (Fig. 2A &B). The measurement starts from the bulbosity of the crown of adjacent second molar because it affects the difficulties and accessibility when utilizing an elevator and rotary hand-piece in the buccal or palatal direction. The IMTM would be further divided into partial buccal position, middle position, and partial palatal position, which we named as the Sun and Zhao Classification. The IMTM was not visible clinically (Figs. 1C and 2 C).

### Local anesthesia

Greater palatine nerve block (Figs. 1D and 2D) and posterior superior alveolar nerve block were administered(Figs. 1E and 2E).

### The procedure of the palatal approach

① Incision design: the main principle is to provide convenience and as least invasive as possible. An envelope flap was designed to avoid injuries to the greater palatine neurovascular bundles. The extent of flap elevation varies according to the visibility of the impacted maxillary third molar. The incision begins at the maxillary tuberosity and extends as far as the distal aspect of the second molar, continuing palatally along the cervical lines of the last two or three teeth, and ending at the mesial aspect of the first molar or second premolar. (Figures 1F and 2 F).

② Bone guttering: Following mucoperiosteal flap elevation, palatal or distal bone guttering was performed using piezosurgery(Fig. 1G) or surgical bur(Fig. 2G) based on the extent of the bony impaction.

③ Tooth luxation: The fulcrum of the dental elevator was positioned at the mesio-palatal most of the IMTM (Fig. 2H), care should be taken to not exert force on the adjacent tooth. After the tooth was luxated (Figs. 1H and 2I), dental forceps were used to deliver the tooth (Figs. 1I and 2 J).

④ Closure: Following tooth extraction, the socket was assessed for oroantral communication prior to hemostasis and primary closure with Silk 3 − 0 suture. (Fig. 1J). (Figures 1K and 2 K).

### The procedure of the buccal approach

Most of the procedures and precautions are the same as palatal approach, the only differences are:

① Incision design: The incision begins at the maxillary tuberosity and extends as far as the bucco-distal aspect of the second molar, continuing obliquely upwards and anteriorly (relieving incision) to the vestibular fold. The flap extension depends on the visibility of the IMTM.

② Bone guttering: Following mucoperiosteal flap elevation, buccal or distal bone guttering was performed.

③ Tooth luxation: A curve elevator should be used with the fulcrum positioned at the bucco-mesial most of the IMTM.

The positions, diagnosis, dental medical history, and radiographic examination of teeth were recorded before the treatment. The operation time was documented from the start of the incision to the luxation of the teeth. Unfavorable tooth fractures, fractures of the alveolar process, intraoperative hemorrhage, root displacement, traumatic ulcer at the commissure of lips, and other complications were also recorded. The post-operative review involved the assessment of the discomfort score of the angle of mouth, and mouth opening using the visual analog scale (VAS), which scores from 0 (no discomfort) to 10 (most uncomfortable). The incidence of alveolar osteitis and postoperative hemorrhage were also documented during the review visits (Fig. 1L).

The statistical analysis was conducted by SPSS 26.0 and a *P*-value of < 0.05 was considered statistically significant. Fisher’s exact test served to explore the differences in enumeration data between the two groups. Mann-Whitney U test was used for the comparison of VAS scores and operation times between the two groups.

The predictor variable is the surgical approach (either buccal or palatal approach), while the outcome variables included operation time, unfavorable tooth fractures, fractures of the alveolar process, intraoperative hemorrhage, root displacement, traumatic ulcer at the commissure of lips, and the pain score of the angle of mouth and mouth opening.

In this study, the covariates are the patients’ age and gender.

## Result

The study was first registered on 19/11/2020 under the Chinese Clinical Trial Registry (registration number is ChiCTR2000040063), and conducted in the outpatient clinic of the Department of Oral and Maxillofacial Surgery, Hospital of Stomatology, Wuhan University. To sum up, 40 patients with 40 IMTMs were enrolled in our study. There were 23 males and 17 females (57.5% and 42.5%, respectively) with a mean age of 29.7 ± 4.3 years old. The demographic information in accordance with 40 teeth were summarized in Table 1. 80% of cases were extracted prior to orthodontic treatment, while 20% of the cases were extracted due to patients’ complaints. In this study, 50% (20 out of 40) were impacted left and right maxillary third molars respectively. All the surgeries were performed by a senior doctor. The first patient enrolled was allotted to the buccal approach arm, while the second patient recruited was assigned to the palatal approach arm. The likewise manner was applied to the subsequent patients enrolled in the study.

**Table 1:** Basic clinical characteristics of 40 teeth

		Total			Buccal Approach			Palatal Approach	
		N = 40			N = 20			N = 20	
		Case number	%		Case number	%		Case number	%
Gender									
	Male	23	57.5		11	55.0		12	60.0
	Female	17	42.5		9	45.0		8	40.0
Age (mean ± SD)		29.7 ± 4.3			28.8 ± 4.1			30.6 ± 4.5	
	< 30	17	42.5		9	45.0		8	40.0
	≥ 30	23	57.5		11	55.0		12	60.0
Tooth position									
	Left	20	50.0		10	50.0		10	50.0
	Right	20	50.0		10	50.0		10	50.0

The operation times were significantly longer in the buccal approach group as compared to the palatal approach group (22.30 ± 5.55 min vs. 13.3 ± 2.76 min, *P*<0.001). The incidence of traumatic ulcers of the lips was significantly higher in the buccal approach group than in the palatal approach group (7/20 vs. 0/20, *P* = 0.008).

No significant differences were found between the buccal approach group and palatal approach group in the occurrence of unfavorable tooth fracture (1/20 vs. 1/20, *P* = 1) and maxillary tuberosity fracture (3/20 vs. 1/20, *P* = 0.61). The remaining tooth fragments were not removed in both patients with unfavourable tooth fracture, as it was curved and the fragments measured less than 3 mm. Oroantral communication, postoperative hemorrhage, root displacement, or alveolar osteitis was not reported in both groups. (Table 2)

**Table 2:** Surgical Information and Complications

		Total			Buccal Approach			Palatal Approach			Buccal *VS.* Palatal
		N = 40			N = 20			N = 20			*P*
Operation Time (min, Mean ± SD)		17.8 ± 6.3			22.3 ± 5.5			13.3 ± 2.8			<0.001^a^
Iatrogenic angular cheilitis											
NO		33	82.5		13	65.0		20	100		0.008 ^b^
YES		7	17.5		7	35.0		0	0		
Oroantral communication											
NO		40	100		20	100		20	100		N/A
YES		0	0		0	0		0	0		
Unfavorable tooth fracture											
NO		38	95.0		19	95.0		19	95.0		1^b^
YES		2	5.0		1	5.0		1	5.0		
Maxillary tuberosity fracture											
NO		36	90.0		17	85.0		19	95.0		0.61 ^b^
YES		4	10.0		3	15.0		1	5.0		
Postoperative Hemorrhage											
NO		40	100		20	100		20	100		N/A
YES		0	0		0	0		0	0		
Root Displacement											
NO		40	100		20	100		20	100		N/A
YES		0	0		0	0		0	0		
Alveolar Osteitis											
NO		40	100		20	100		20	100		N/A
YES		0	0		0	0		0	0		

(a) Mann-Whitney U test; (b) Fisher‘s exact test

Generally, the patients felt less discomfort at the commissure of the mouth in the palatal group in comparison to the buccal approach group. There was however no significant difference in discomfort upon mouth opening. (*P*<0.001). The discomfort of mouth opening was mild in both groups without significant difference (*P* = 0.21). (Table 3)

**Table 3:** visual analog scale about discomfort during surgery

			Buccal Approach			Palatal Approach			Buccal *VS.* Palatal
			N = 20			N = 20			*P*
			Median (Q1-Q3)	Mean ± SD		Median (Q1-Q3)	Mean ± SD		
Retraction of commissure of the mouth			7 (6–8)	6.9 ± 1.1		0 (0–1)	0.5 ± 0.5		<0.001 ^a^
Mouth opening			1 (0–1)	0.9 ± 0.7		1 (1-1.75)	1.2 ± 0.5		0.21 ^a^

a. Mann-Whitney U test

## Discussion

IMTM, especially those which are mesioangularly impacted and deeply impacted, may cause external root resorption of the adjacent second molar. Prophylactic removal is recommended for patients with impacted maxillary third molars IMTM is recommended for prophylactic removal for patients over 25 years and with mesially inclined and deeply impacted [12]. Due to the limited visualization and access, surgical removal of an IMTM can be complex for the dental surgeons, which may cause serious complications [13, 14]. Adverse events during surgery are associated with the relationship with the adjacent second molar and the periodontal space [15]. In most textbooks or articles in either Chinese or English, the surgical removal of impacted maxillary third molar surgery is usually described to be performed via a buccal approach. This is feasible if an IMTM were located on the buccal-distal aspect of the second molars according to the principle of proximity. However, it can be more difficult to perform the surgery from a buccal approach if the crown of IMTM were distally or palatally impacted to the adjacent second molars as the operating field would be blocked. As a result, greater retractive force maybe required and iatrogenic pressure applied intra-operatively by the instruments may lead to traumatic ulcers on the lips. For a better view, the angle of the mouth should be retracted, as well as the pressure exerted by the tooth extraction equipment, which may lead to traumatic ulcers to the commissure of the lips. Here, we presented the use of palatal approach for surgical removal of such IMTM with higher efficiency, safer, and a generally better patient experience.

Archer et al. classified IMTMs into mesioangular, distoangular, vertical, horizontal, buccoangular, linguoangular, or inverted [16, 17]. It is classified as Class A, B, and C according to the depth of impaction in relation to the second molar. Class B is defined as the occlusal surface of the impacted tooth is located in the middle of the crown of the adjacent second molar. Only Class B IMTM were enrolled in our study, as it is unnecessary to raise the palatal flap to extract Class A IMTM. While for class C IMTM, the bony impaction is the deepest, therefore it may be impractical to compare surgical approaches because of the close relationship of the IMTM with the adjacent tooth, and maxillary sinus [18]. Although the classification is classical, the classification lacks a description of the buccal-palatal relationship between the maxillary third molar and second molar. CBCT allows visualization of the occlusal surface position of IMTM, and the buccal-palatal relationship between the maxillary third molar and second molar. We proposed a new classification based on the buccal-palatal relationship between the maxillary third molar and the second molar shown on the CBCT: partial buccal position, middle position, and partial palatal position. Similar to the previous study [16], the crowns of the IMTM were not completely covered by the alveolar bone shown in CBCT. It is only visible after the flap was elevated.

According to the proximity principle, the surgeon should apply a nearer and safer approach. The buccal approach might be inappropriate, especially in cases with a buccally inclined second molar and maxillary arch that curved palatally at the second molar region. In our study, palatal approach surgery is more efficient for such IMTMs, in which the crowns were located in the distal or palatal aspect of the second molar, and the occlusal surface was positioned palatally. The gist of the surgery is to identify the fulcrum, which is located at the mesial most of IMTM. It is relatively safe to use piezosurgery for bone guttering in both approaches. However, when using a fissure bur, provides a better surgical view and is easier to protect soft tissue in palatal approach surgery.

CBCT allows the surgeon to measure the thickness of the bone covering the tooth and design the luxation direction of the IMTM. As to the palatal approach to IMTM surgery, all the teeth were luxated palatally, but not only limited to the palatal aspect of the IMTM. In 50% of cases performed using the palatal approach, the buccal alveolar ridge was used as a fulcrum for luxation palatally. The software we used in this study allows segmentation of the teeth from the alveolar bone using Artificial Intelligence (AI) technology, which showed a better view and therefore provides precise decisions.

As to the mesioangular IMTM, tooth sectioning was not performed for several reasons. In contrast to the impacted mandibular third molars, the surgical field to perform the tooth sectioning is not as good either in buccal or palatal approaches. In addition, the depth of the tooth section is difficult to control, and as a result, it may disrupt the maxillary sinus if tooth sectioning was performed excessively. Furthermore, incomplete tooth sectioning might cause loss of the fulcrum and increase the difficulty of the surgical removal. To preserve at least 5 mm of the alveolar crest, piezosurgery or fissure bur is used to remove the bone resistance in the direction of luxation. The IMTM could be luxated palatally, posteriorly, outwards, and downwards.

As shown in our study, the total operating time for the palatal approach is significantly shorter than the buccal approach. Although the palatal approach may require wider mouth opening, the patients only experienced mild discomfort as the duration of the surgery was relatively shorter. To our knowledge, it is the first study to introduce a palatal approach to extract IMTM and reduce the incidence of traumatic ulcers on lips following tooth extraction.

In previous study, the usage of the palatal approach may be risky and difficult in surgical removal of these teeth without showing a clear description of the surgical techniques [10]. The palatal approach needs considerable care to prevent tearing of the flap and damage to the greater palatine neurovascular bundle which can lead to significant intra-operative and post-operative complications [19, 20]. M Butler et al. declared the difficulty of this procedure is further compounded by poor access to the surgical site without any direct evidence [10, 19].

Nevertheless, there are some limitations in our study. To date, there are limited publications illustrating alternative surgical techniques in the removal of such impacted maxillary third molars, thus further comparison or discussion may be limited. The Class C cases were not enrolled in our study for the variable position. Whether this strategy is advised in deeper class C cases is unknown. As this is a pilot study, we did not randomly divide the patients, and a randomized controlled trial will be carried out for the subsequent study.

To sum up, our study demonstrated a new strategy for the surgical removal of IMTM. It is more efficient to perform surgery with a palatal approach if a Class B mesioangularly IMTM is located in the non-buccal aspect of the adjacent second molar, which avoids traumatic ulcers of the lips.

## Data Availability

All data generated or analyzed during this study are included in this published article.
